# Diversity of gene expression responses to light quality in barley

**DOI:** 10.1038/s41598-023-44263-8

**Published:** 2023-10-10

**Authors:** Álvaro Rodríguez del Río, Arantxa Monteagudo, Bruno Contreras-Moreira, Tibor Kiss, Marianna Mayer, Ildikó Karsai, Ernesto Igartua, Ana M. Casas

**Affiliations:** 1 Department of Genetics and Plant Breeding, Aula Dei Experimental Station, CSIC, Avda Montañana 1005, 50059 Zaragoza, Spain; 2 Centro de Biotecnología y Genómica de Plantas, UPM/INIA-CSIC, Madrid, Spain; 3 Fundación ARAID, Zaragoza, Spain; 4 Centre for Agriculture Research ELKH (ATK), Martonvásár, Hungary; 5 Center for Research and Development, Food and Wine Center of Excellence, Eszterházy Károly Catholic University, Eger, Hungary

**Keywords:** Light responses, Natural variation in plants, Plant development

## Abstract

Light quality influence on barley development is poorly understood. We exposed three barley genotypes with either sensitive or insensitive response to two light sources producing different light spectra, fluorescent bulbs, and metal halide lamps, keeping constant light intensity, duration, and temperature. Through RNA-seq, we identified the main genes and pathways involved in the genotypic responses. A first analysis identified genotypic differences in gene expression of development-related genes, including photoreceptors and flowering time genes. Genes from the vernalization pathway of light quality-sensitive genotypes were affected by fluorescent light. In particular, vernalization-related repressors reacted differently: *HvVRN2* did not experience relevant changes, whereas *HvOS2* expression increased under fluorescent light. To identify the genes primarily related to light quality responses, and avoid the confounding effect of plant developmental stage, genes influenced by development were masked in a second analysis. Quantitative expression levels of *PPD-H1*, which influenced *HvVRN1* and *HvFT1,* explained genotypic differences in development. Upstream mechanisms (light signaling and circadian clock) were also altered, but no specific genes linking photoreceptors and the photoperiod pathway were identified. The variety of light-quality sensitivities reveals the presence of possible mechanisms of adaptation of winter and facultative barley to latitudinal variation in light quality, which deserves further research.

## Introduction

Plant development is governed by environmental stimuli. One of the main developmental triggers is light, whose features (light duration, quantity, and quality) regulate growth and determine environmental adaptation^1,2^.

Light quality and intensity are not constant in natural environments, varying throughout days, seasons, climates, and atmospheric conditions^3,4^. In fact, responses of plants to light features have been thoroughly analyzed^1,2,5–7^. However, there is little research on the existence of genetic diversity in responses to light quality in annual crop plants^2^. Through the years we observed remarkable genotypic differences in barley development when tested in different types of growth chambers, using various light sources. Motivated by this, we explored the phenotypic variability of eleven barley genotypes in response to different light quality environments under fully inductive conditions of flowering (saturated vernalization requirement, long photoperiod and optimal ambient temperature), and found striking genotypic differences^8^. In that study, plants were exposed to two light sources, producing the same light intensity, fluorescent light, and metal halide bulbs. Although the light source was the only differential factor between treatments, plant development was delayed under fluorescent light compared to metal halide light. This delay, however, was far from homogeneous. We found diverse phenotypic responses, and classified the varieties into light quality-sensitive (strongly delayed development under fluorescent light) and light quality-insensitive (almost similar development pace under the two light conditions).

The combined effect of light duration and temperature is critical for crop development^9^. Both are particularly important in winter cereals, which need to experience a cold period before the spring (a process known as *vernalization*), when long days (LD) trigger flowering. Two main genes control the vernalization response in winter cereals. In barley (*Hordeum vulgare* L.), these genes are *HvVRN1* and *HvVRN2*^10,11^, which interact with the floral pathway integrator *HvFT1*^12^. When the cold requirement has not been satisfied, long-days promote *HvVRN2* expression, repressing *HvFT1*, and delaying flowering until plants complete vernalization^13,14^. Only when cold induces *HvVRN1*, *HvVRN2* is repressed, and together with the influence of LD, promote the expression of the flowering integrator *HvFT1*. In winter wheat and barley, there is a strong photoperiod response, mediated by the *PPD1* genes, which occurs only when plants have received sufficient vernalization. *PPD-H1* also has an inductive effect on *HvFT1* expression.

In this study, we aim at identifying the genetic pathways underlying the unexpected different sensitivities of the barley genotypes to light quality identified in the previous work^8^. We analyze the variation in gene regulation underlying the contrasting responses to two light spectral conditions (produced by fluorescent and metal halide lamps) of three barley genotypes which showed striking phenotypic responses ranging from insensitive to sensitive to light quality. The presence of genotypic diversity hints at a potential adaptive role of these responses. Our final aim is to contribute to unraveling the diversity and adaptive nature of the responses of cereals to environmental cues, focusing on the relatively new target of light quality.

## Results

From the set of 11 barley varieties described previously^8^, we selected three (Esterel, Price, and WA1614-95), which cover the full range of responses observed in that study. In particular, Esterel was scored as moderately sensitive to light quality (from now on, we will designate it as insensitive, for simplicity), Price as intermediate, and WA1614-95 as highly sensitive to light quality.

After vernalization, plants from these genotypes were at a similar developmental stage (Z11-Z12 of Zadoks’s scale^15^). Subsequently, the plants were transferred to two growth chambers, either with white fluorescent (hereafter fluorescent, **F**) or metal halide (hereafter metal, **M**) light bulbs as explained in Materials and Methods.

**Figure 1:**
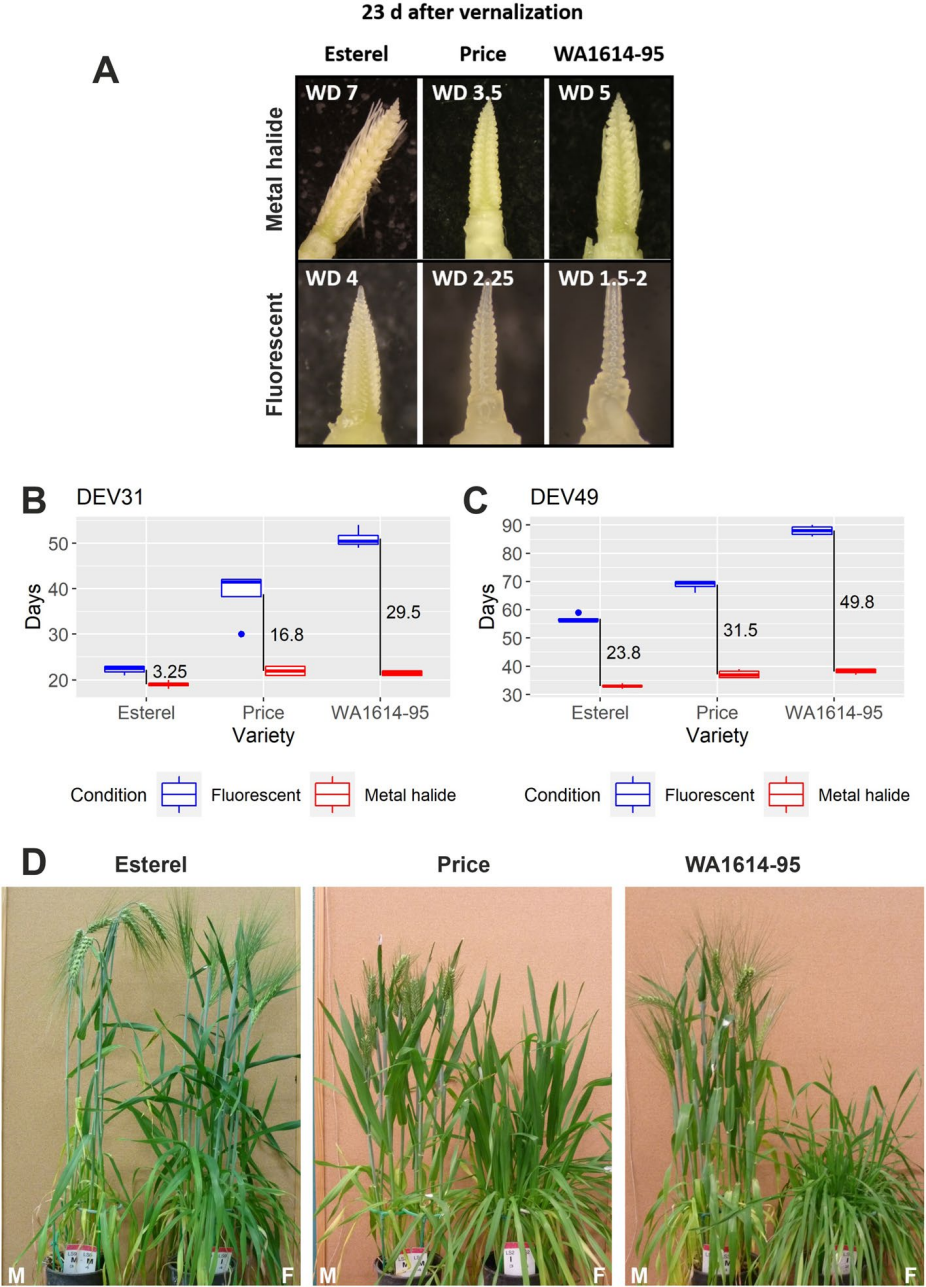
Phenotypic differences between varieties. (**A**) Apex development in plants dissected 23 days after the end of the vernalization treatment. *WD* Waddington stage. (**B**) and (**C**) Days to first node (DEV31) and awn appearance (DEV49), expressed in days from the end of the vernalization treatment, measured in 4 biological replicates. Vertical black lines represent the days of difference between fluorescent and metal halide light conditions. (**D**) Plants photographed 58 days after the end of the vernalization treatment. Data in panel (**B**) adapted from^8^.

Plants developed normally under M conditions. Duration of developmental phases and relative differences between the genotypes responded to expectations, based on our previous knowledge of these genotypes, including field observations. However, under F conditions, plant development was delayed, with uncommonly long periods until the appearance of the first node, and from there until the onset of the rapid stem elongation phase, although genotypic responses differed vastly (see Fig. 1). Therefore, the metal halide light source (M), which elicited “normal” responses, was considered as control for the RNA-seq analysis.

### RNA-seq experiments

We measured gene expression in three biological replicates of each genotype grown under the two light conditions. Sequencing of the 18 samples produced a total amount of 1.92 billion paired-end reads. The joint de novo assembly for the three genotypes contained 375,488 isoforms, from which we obtained 181,337 unigenes. One sample (Esterel_F2) showed outlier correlation coefficients of transcript abundance with the other two biological replicates (see Fig. S1B,D) and was subsequently discarded from downstream analyses. Price and WA1614-95 expression patterns were highly correlated in metal halide light (M) conditions, consistently for all replicates, indicating similar responses (Fig. S1A). However, the correlations among genotypes were lower in fluorescent light (F) conditions (Fig. S1B), indicating larger variability of responses. The insensitive genotype Esterel showed lower correlation coefficients with the other genotypes in both conditions, probably a combination of a distinct reaction to light quality and its faster development.

### Analysis of gene expression

We observed differentially expressed (DE) genes that behaved similarly in the three genotypes, whereas others presented different patterns among genotypes. The first type points at genes that may be intrinsically involved in response to light quality, or to genes whose expression is influenced by plant development. The second indicates genes likely related to different genotypic responses to light quality.

**Figure 2:**
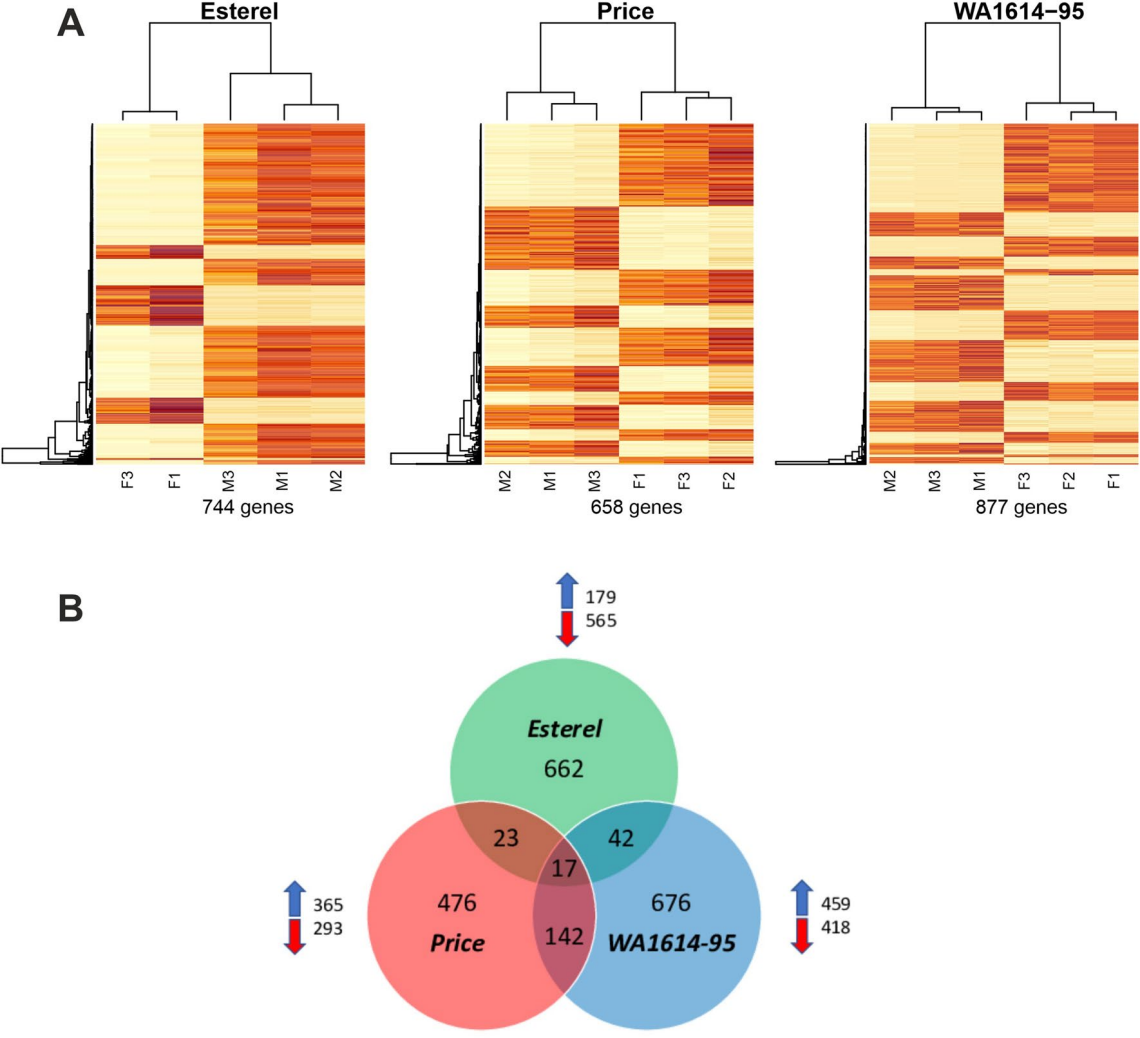
Differentially expressed (DE) genes in the three genotypes (q-value < 0.01). (**A**) Clustering of DE genes with Morex CDS sequences used as reference. Colour code: red, upregulated in fluorescent light; yellow, downregulated. Three biological replicates per variety and condition are represented, except for Esterel in fluorescent light, from which a replicate was discarded. *F* fluorescent; *M* metal halide. (**B**) Venn diagram showing the intersection in the number of DE genes among genotypes. Blue and red arrows indicate the number of DE genes upregulated or down regulated in fluorescent conditions in each genotype.

Within genotypes, DE genes in Esterel were predominantly down-regulated (in fluorescent compared to metal), whereas Price showed more up-regulated than down-regulated genes, and WA1614-95 showed a similar number of up- and down-regulated DE genes (Fig. 2).

**Figure 3:**
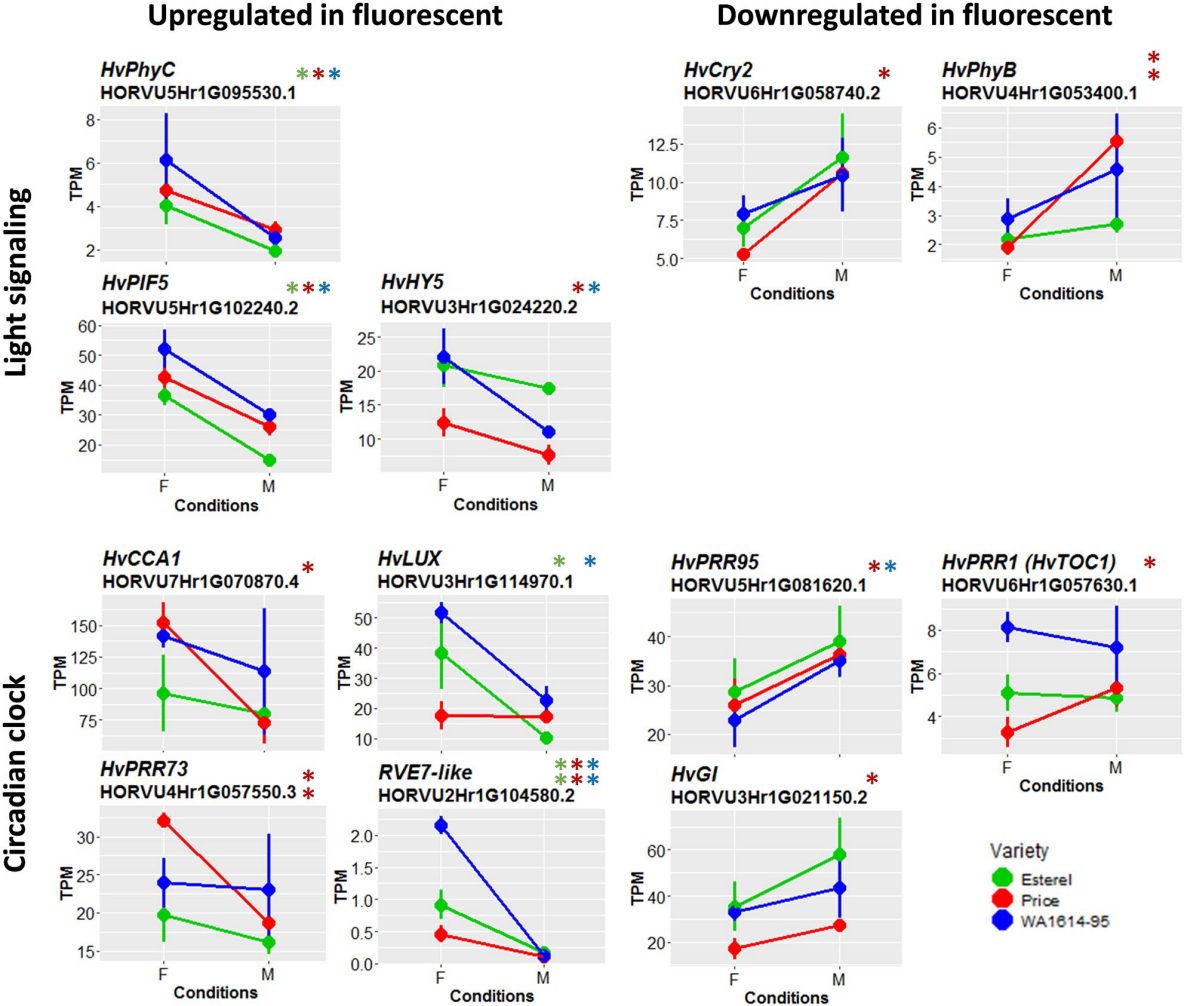
Expression levels of light signaling and circadian clock selected genes in the three genotypes. Differences between treatments are significant at q-value < 0.05 (*) or q-value < 0.01 (**). Genes are separated in two groups: upregulated (left) and downregulated (right) under fluorescent conditions. Genotypes are coded as green (Esterel), red (Price) and blue (WA1614-95), in fluorescent (F) or metal halide conditions (M). *TPM* transcripts per million.

**Figure 4:**
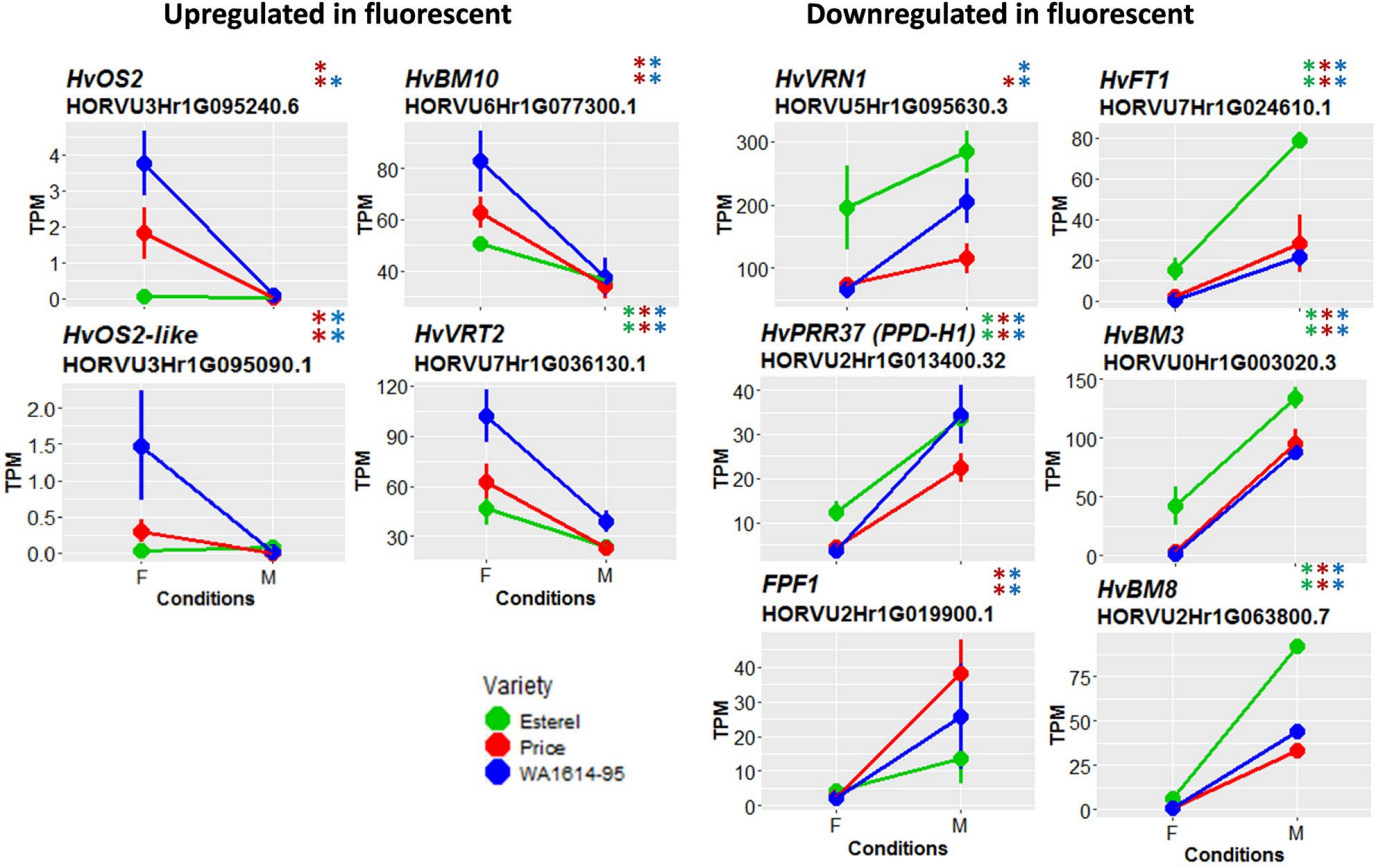
Expression levels of development-related genes in the three genotypes. Genes are separated in two groups: upregulated (left) and downregulated (right) under fluorescent conditions. F, fluorescent conditions; M, metal halide conditions. Genotypes coded as in Fig. 4. Differences between treatments are significant at q-value < 0.05 (*) or q-value < 0.01 (**). *TPM* transcripts per million.

The intersection of DE genes for the three genotypes comprised 17 sequences (Table 1). Sixteen of them showed the same direction of variation in the three genotypes. Among them, as expected due to the growth stage differences among genotypes at the moment of sampling, there was a relative abundance of genes involved in plant development, like MADS box TFs *HvBM3* and *HvBM8*^16,17^, the pseudo-response regulator *PPD-H1* (*HvPRR37*^18^), *HvFT1* (*Flowering locus T*^12,19^) were down-regulated under fluorescent light conditions. Conversely, short vegetative phase MADS box TF *HvVRT2* (*vegetative to reproductive transition 2*^20,21^) and *RVE7*-like, a MYB family TF *circadian 1*, involved in circadian regulation in Arabidopsis (Ref.^22^, were up-regulated in fluorescent light. *RVE7*-like and *HvVRT2* were expressed at higher levels in sensitive WA1614-95, whereas *HvBM3*, *HvBM8*, and *HvFT1* showed higher expression in the insensitive line, Esterel (Figs. 3, 4).

**Table 1:** List of DE genes (q-value < 0.01) shared by three barley varieties studied. We show up-regulated (u) and down-regulated (d) genes in fluorescent light for each genotype.

Target ID Morex v1.0^a^	Target ID Morex v3.0^b^	Description^b^	Gene	Cite	Price	WA1614-95	Esterel
*HORVU0Hr1G003020.3*	*HORVU.MOREX.r3.2H* *G0127410.1*	*MADS box transcription factor*	*HvBM3*	^16,17^	d	d	d
*HORVU2Hr1G063800.7*	*HORVU.MOREX.r3.2H* *G0156870.1*	*MADS box transcription factor*	*HvBM8*	^16,17^	d	d	d
*HORVU2Hr1G063810.1*					d	d	d
*HORVU7Hr1G036130.1*	*HORVU.MOREX.r3.7H* *G0664320.1*	*MADS box transcription factor*	*HvVRT2*	^20,21^	u	u	u
*HORVU7Hr1G083670.3*	*HORVU.MOREX.r3.7H* *G0713370.1*	*Cytochrome P450 family protein*			u	u	u
*HORVU2Hr1G013400.32*	*HORVU.MOREX.r3.2H* *G0107710.1*	*Pseudo-response regulator*	*HvPRR37 (PPD-H1)*	^18^	d	d	d
*HORVU4Hr1G090860.12*	*HORVU.MOREX.r3.4H* *G0418190.1*	*Metacaspase-1*	*cell death*		u	u	u
*HORVU5Hr1G071940.2*	*HORVU.MOREX.r3.5H* *G0489730.1*	*Glycosyltransferase*			u	u	u
*HORVU2Hr1G024120.10*	*HORVU.MOREX.r3.2H* *G0118180.1*	*Terpene synthase*			d	d	d
*HORVU0Hr1G038850.2*	*HORVU.MOREX.r3.6H* *G0622200.1*	*Protein kinase*			u	u	u
*HORVU3Hr1G111550.2*	*–*				u	u	d
*HORVU3Hr1G021880.1*	*HORVU.MOREX.r3.3H* *G0239170.1*	*Glycosyltransferase*	*Glycosyl-transferase*		d	d	d
*HORVU3Hr1G087100.1*	*HORVU.MOREX.r3.7H* *G0653910.1*	*Flowering locus T*	*HvFT1 (VRN-H3)*	^12,19^	d	d	d
*HORVU7Hr1G024610.1*					d	d	d
*HORVU5Hr1G029260.1*	*HORVU.MOREX.r3.5H* *G0449270.1*	*Protein kinase family protein*			d	d	d
*HORVU2Hr1G104580.2*	*HORVU.MOREX.r3.2H* *G0196050.1*	*Homeodomain-like superfamily protein*	*RVE7-like*	^22^	u	u	u
*HORVU1Hr1G076460.3*	*HORVU.MOREX.r3.1H* *G0077260.1*	*Hemoglobin*		^23^	u	u	u

^a^Morex reference genome v1.0 ^63^.

^b^Morex reference genome v3.0 ^66^.

The only DE gene with opposing trend between the sensitive and insensitive genotypes at P < 0.01 was a 2176b unigene mapped to reference gene *HORVU3Hr1G111550.2*. Transcripts encoded by this gene have been reported in seedlings in day and night in cultivar Haruna Nijo^24^ and Barke^25^, but otherwise lack any functional annotation, likely due to this gene being incomplete in the reference cultivar Morex. However, the transcript shows perfect alignment with ten genotypes represented in the barley pangenome^26^, such as Igri, Barke, Golden Promise, RGT Planet and others (two stretches of 126 bp and 2048 bp, from 99.2 to 100% identity, see Fig. S2). Therefore, there is genetic variation for this gene in the barley germplasm apparently involved in the differential response to light. This gene is a relevant target for future research.

When we looked into DE genes shared by just two varieties (Fig. 2, Supplementary Datasets S1, S2), the intersection between the two lines sensitive to light quality, Price and WA1614-95, presented the largest number of DE genes (142), all in the same direction (74 up-regulated and 68 down-regulated in F), indicating commonality of responses. In the intersection of insensitive Esterel and sensitive Price, only 20 genes showed differential expression with similar sign (14 up and 6 down), and 3 genes presented opposite directions (up-regulated in Price and down-regulated in Esterel). The intersection between insensitive Esterel and sensitive WA-1614-95, the two genotypes with most contrasting responses, also showed the highest number of DE genes varying in opposite directions (29, vs only 13 in the same direction). WA1614-95 had more up- (35) than down-regulated (7) genes, whereas Esterel had more down- (32) than up-regulated (10). These results suggest divergence of gene expression responses to the F light for the three genotypes, compatible with the phenotypic responses. In the intersection between Price and WA1614-95, a Flowering Promoting Factor gene was down-regulated in fluorescent light whereas transcripts for a receptor-like kinase, a RING/U-box superfamily protein, MADS-box, myb-domain or WRKY TFs, and several transcripts coding for jasmonate-induced proteins were up-regulated in fluorescent light. DE genes in the intersection between WA1614-95 and Esterel included two WRKY TFs (Supplementary Datasets S1, S2).

### Identifying genes affected by the primary response to light source

As seen previously, gene expression comparisons across genotypes and, specially, across light sources, were influenced by the developmental stage of plants. Esterel in M light developed faster than the other genotypes and, at the sampling time of 20 days, it had reached first node appearance (DEV31) the previous day, whereas Price and WA1614-95 were comparatively delayed. According to the apex morphological features they had not reached DEV31 on the sampling date (Fig. 1). Under F light, none of the genotypes reached DEV31 on sampling day, although Esterel presented a more advanced stage than the other two genotypes. Therefore, we can expect that differences in gene expression between light sources for the three genotypes, and particularly for Esterel, could be partially due to the expression of genes specific of developmental phases, as the genotypes were sampled in different phenological phases.

In consequence, the sets of DE genes found between M and F lights are a mixture of genes responding directly to light, and genes that act downstream, related to the delay in development (caused by exposure to fluorescent light, but not as a primary response). We have attempted to differentiate the former by identifying and masking the latter in the M-F comparisons. The sets of DE genes due exclusively to development, and not to light, were identified by comparing the DE genes between the more developmentally advanced Esterel and less advanced Price and WA1614-95, exclusively in M light (therefore, with no involvement of light quality). Besides intrinsic genotypic differences, some DE genes in those two comparisons will be due to the different phenological stage between Esterel and the other two genotypes (Fig. 1). DE genes (and GOs, see below) shared between the two comparisons (Esterel-Price and Esterel-WA1614-95) were declared as development-related, likely independent of light source. These genes were then masked in the comparisons between M and F lights for each genotype. Masking was done when a transcript was DE in both comparisons Esterel-Price and Esterel-WA1624-95 in M light. The DE genes left after masking were considered as the most likely related with the primary response to the light treatments. For the sensitive varieties Price and WA1614-95, 455 (58.4%) and 403 (35.8%) DE genes were kept, respectively (out of 779 and 1126), after masking (Table 2). For the insensitive variety Esterel, interestingly, only 122 DE genes (5.2%) were kept after eliminating genes affected by development (out of 931). This is an indication that, after removing the DE genes due to the samples being at different developmental stages, the insensitive cultivar presented three times less DE genes affected by light, and independent of development, than the intermediate and highly sensitive ones. This observation is consistent with the expected behavior of an insensitive (or less responsive) *vs* the two sensitive genotypes.

**Table 2:** Differentially expressed genes (DE, q-value < 0.01), before (column Total) and after (column Light) masking genes whose behavior could be ascribed to differences that were related to development (column Development).

Category	Total	Development	Light
DE esterel	931	809	122
downF	708	671	37
upF	223	138	85
DE price	779	324	455
downF	344	143	201
upF	435	181	254
DE WA1614-95	1126	723	403
downF	576	410	166
upF	550	313	237

In addition, we explored the expression of genes known to be involved in light perception (phytochromes, cryptochromes), circadian clock, flowering initiation, and development. For this purpose, we extended the search to DE genes with q-value < 0.05 (Figs. 3, 4). Among these, *HvPhyC* was up-regulated under fluorescent light in the three genotypes, whereas *HvPhyB* and *HvCry2* were only up-regulated in metal in Price. Only the latter was apparently related to a primary response to light quality. The MADS-box TF *HORVU3Hr1G095090* (similar to *TaFLC-A4-2*, as described in^27^) displayed contrasting patterns between the two sensitive (up-regulated) and insensitive (unchanged) genotypes, apparently as a primary response to light.

Masking discarded some of the development-related genes like *HvBM3*, *HvBM8*, *HvVRT2*, or *HvFT1* (*VRN-H3*) as direct responses to light quality. Their differential expression likely occurred downstream of the primary responses. On the other hand, some genes had a striking difference in expression between M and F, in the same direction, but with quantitative differences closely resembling the differences in sensitivity of the genotypes. Transcripts *HORVU.MOREX.r3.2HG0107710.1* (corresponding to pseudo-response regulator *HvPRR37*, also known as *PPD-H1*), *HORVU.MOREX.r3.4HG0418190.1* (metacaspase-1), and *HORVU.MOREX.r3.3HG0239170.1* (glycosyltransferase) showed extreme changes in expression in WA1614-95, intermediate in Price, and low (but still highly significant) in Esterel. *PPD-H1* is known to be involved in light responses, and the other two genes could be in close connection to it.

### GO enrichment analysis

We carried out a GO enrichment analysis to find functional commonalities among the genes present in each of the three sets of within genotype DE genes. The GO terms associated with DE genes are listed in Supplementary Dataset S8, using two q-value cutoffs, 0.05 and 0.01. The GO terms enriched in each genotype were in many cases due to differences in development among plants grown in fluorescent light (see Supplementary Dataset S9). After masking the 27 GOs related to plant development, following the same procedure as in the previous section, only 7 categories were left in the Esterel M-F comparison (see Table 3). Nine and two GO were enriched in the M-F comparisons for Price and WA1614-95 after the masking, respectively (none in common), indicating specific responses of those genotypes. Out of the 7 outstanding GOs in Esterel, three were related to the development of sexual organs, probably because they were not captured among the genes affected by development, as Esterel in metal halide light was the only genotype reaching floret formation stages. The three GO categories left may contain some of the genes primarily responsible for the response to light in Esterel. Among the GO found in Price, three were related to tetrapyrroles and porphyrin, molecules at the core of chlorophyll and hemoglobin.

**Table 3:** Enriched GO terms associated to differentially downregulated genes (DE) in F light.

Genotype	GO term	Significance
Esterel		
GO:0006487	Protein N-linked glycosylation	*
GO:0008037	Cell recognition	**
GO:0009856	Pollination	*
GO:0009875	Pollen-pistil interaction	**
GO:0044706	Multi-multicellular organism process	**
GO:0048544	Recognition of pollen	**
GO:0051704	Multi-organism process	**
Price		
GO:0033013	Tetrapyrrole metabolic process	*
GO:1901566	Organonitrogen compound biosynthetic process	*
GO:0033014	Tetrapyrrole biosynthetic process	*
GO:0019752	Carboxylic acid metabolic process	*
GO:0043436	Oxoacid metabolic process	*
GO:0006520	Cellular amino acid metabolic process	*
GO:0006082	Organic acid metabolic process	*
GO:0044281	Small molecule metabolic process	*
GO:0006778	Porphyrin-containing compound metabolic process	*
WA1614-95		
GO:0043094	Cellular metabolic compound salvage	*
GO:0015977	Carbon fixation	*

Asterisks indicate significant enrichment with associated Q-values < 0.05 (*) and < 0.01 (**).

### Discovery of regulatory motifs upstream of DE genes

To get more insight into the mechanisms that could be responsible for the primary response to light quality, we analyzed the proximal promoters of up-regulated and down-regulated genes in Price and WA1614-95, after removing genes affected by development (see above). The hypothesis was that DE genes between M and F for these two genotypes might share common regulatory motifs that would explain their expression patterns. In total there were 50 DE genes in common for the two genotypes, all showing variation in the same direction, with 19 down-regulated and 31 up-regulated in F light (Supplementary Dataset S1). Motif analysis found a putative regulatory motif with consensus AAATACAt built out of 40 and 28 sites within [− 500, + 200] and [− 500, 0] sequences of down-regulated genes, respectively (see Fig. S3). This motif is not significantly similar to any plant motif in the footprintDB collection, although it does resemble a homeobox-type motif. Among the transcription factors in the list of DE genes, none was predicted to recognize this motif (see Table S3).

## Discussion

Light-sensitive and insensitive varieties showed strikingly different patterns of DE genes. However, there was a remarkable similarity in the sets of DE genes for sensitive varieties WA1614-95 and Price, not only in genes related to development, but also in those whose expression was more likely affected by light quality.

The delayed development of plants grown in fluorescent light was paralleled by dramatic changes in the expression of development-related genes, including several MADS-box genes. This was expected, given that RNA samples were taken from plants at different growth stages, due to the marked developmental delay induced by the fluorescent light. Two major flowering time genes, *PPD-H1* and *HvFT1*, were also down-regulated in fluorescent light. All these genes are crucial for barley development. The lesser induction of *PPD-H1* in fluorescent light could explain the downregulation of most genes related with development, like *HvBM3* and *HvBM8*, because it mediates the long-day induction of *HvFT1*^18^, which acts upstream of those genes. Moreover, several studies have reported the *PPD-H1*-dependent up-regulation of *HvBM3* and, *HvBM8* during development^17,28,29^.

The differential expression of *FLC*-like *HvOS2* and its paralog *HORVU3Hr1G095090* in the two sensitive genotypes indicated a specific response to light quality that could be related with the vernalization process. In wheat, the duplication within the *FLC*-clade has been related to plant adaptation^27^. *HvOS2* represses the expression of *Flowering Promoting Factor1*-like genes (*FPF1*-like)^30,31^, which also appear differentially expressed in our study (Fig. 4). *HvOS2* expression responds to cold, mediated by *HvVRN1*^32^, which was also differentially expressed only in the sensitive varieties. This last gene should have been fully induced after vernalization in all three varieties (even more so in WA1614-95, which needs little vernalization), but it was clearly less induced in fluorescent light in Price and WA1614-95. The *HvOS2* paralog encodes a MADS-box TF, *FLC-like,* related to *TaFLC-A2* and *TaFLC-B2*, genes that interestingly show differential expression in *phyB* and *phyC* wheat mutants, under long day photoperiods^33^. Adding to the temperature-like effect of the fluorescent light, genes related with cold acclimation and vernalization as *HvVRT2*^20,34^, showed consistent higher expression under fluorescent light. Upregulation of repressors and cold-induced genes under fluorescent light in fully-vernalized plants and LD, indicates that these plants are not sensing the favorable conditions, and remain in the cold acclimation phase, eliciting cold-related responses, particularly in the sensitive varieties. These results suggest a light-mediated de-vernalization, similar to the processes induced by light intensity, heat or chemical compounds^35–37^. However, a reversion of vernalization was not confirmed by the performance of *HvVRN2*, which did not show a clear recovery of expression in Price and Esterel under F light^8^. Furthermore, WA1614-95 lacks the *HvVRN2* gene and, therefore, its vernalization need due to the presence of a winter *Hvvrn1* allele is minimal. This different effect of light quality on the two known flowering repressors whose action is relieved by vernalization, points at possible differences in their regulation, beyond the control exerted by *HvVRN1*. *HvOS2* expression seemed affected by light quality, whereas *HvVRN2* was not. This is another result that deserves further research, as there is a large knowledge gap on the functioning of *HvOS2* in barley and wheat. Therefore, we conclude that, although the vernalization pathway is affected by light quality, the responses found cannot be fully explained by them, and there must be other pathways involved.

Land plants possess two types of signal-transducing photoreceptors: phytochromes (in cereals PhyA, PhyB, and PhyC) absorbing principally in the 600–800 nm waveband, and cryptochromes (Cry1, Cry2), absorbing only in the 300–500 nm band^4,38^. Both phytochrome and cryptochromes interact with transcription factors (TF) known as Phytochrome Interacting Factors (PIFs)^39,40^, regulating clock and flowering time genes^41^, and are at the top of fundamental light-driven processes. PhyC is responsible for the connection between the photoreceptor and the photoperiod-development pathways, activating the long-day photoperiod response gene, *PPD-1* in LD^42,43^. Phytochromes and cryptochromes perceive light signals and participate in multiple signaling pathways, optimizing plant growth and development^44,45^. Fittingly, there was higher expression levels of *HvPhyC* under fluorescent light in all three genotypes, particularly in sensitive WA1614-95, and the opposite trends for *HvPhyB* and *HvCry2* in the two light treatments (consistent with their antagonist role, reported by^46^. The modified expression of these three signaling genes could be at the top of the cascade of changes in gene expression found in downstream pathways, also observed by^47^.

PhyC and PhyB signals may have cascaded down through PPD1. Indeed, they are known to activate PPD1 in long days, a condition used in our experiment^42,43,48^. Moreover, studies in barley show that natural variation in PHYC underlies differences in daylength sensitivity amongst barley cultivars^42^. We observed alterations of expression levels of *HvPHYC* (in all genotypes) and *HvPHYB* (only significant for Price) in F light. It is thus sensible to think that altered phytochrome expression affected *PPD-H1* and its downstream effects, like a reduced *HvFT1* expression and a delayed transition from vegetative to reproductive stage, precisely what we observe under fluorescent light. This phenomenon was also observed in wheat, also mediated by the circadian clock^43^. Consistent with that hypothesis, we observed some changes in expression of clock genes, particularly in sensitive varieties. Consequently, we hypothesize that the altered expression of PHYC, and possibly of other photoreceptors, produces delays in development (from moderate in Esterel to severe in WA1614-95), by an alteration of the fine balance between active and inactive forms of the phytochrome proteins, which in turn reduces the induction of *PPD-H1* expression.

In winter barley, the dominant *PPD-H1* allele exerts its accelerating effect after complete vernalization, and with daylengths above a certain threshold (approximately 12 h). These conditions were met in our experiment. Therefore, we did not expect differences in *PPD-H1* expression between light treatments, and all three varieties should have reacted equally to day length stimuli. As expected, *PPD-H1* expression was similar in the three varieties, in the M light treatment. This notwithstanding, *PPD-H1* was clearly less expressed under fluorescent light, indicating the presence of limiting factors which were not present under metal halide light. Interestingly, there were quantitative differences in expression among them: in Esterel, it was reduced by a factor of 2.7, whereas in Price and WA1614-95, the factors were 4.9 and 9.3, respectively. These reductions are proportional to the sensitivity of these three varieties to light quality.

Reductions of expression of similar magnitudes were observed for *HvFT1*, the integrator of the photoperiod and vernalization signals that acts downstream of *PPD-H1*, and induces the start of reproductive development in barley. Our hypothesis to explain the large developmental differences observed under the fluorescent light is that *PPD-H1* is the final recipient of the signals from photoreceptors, and its reduced expression affected *HvFT1* induction, causing a developmental delay that was proportional to the level of altered expression of these genes suffered by each variety. We believe that *PPD-H1* is a good candidate, playing a central part in the phenotypic responses observed.

This hypothesis is further supported by relevant findings for the intermediaries between phytochromes and the photoperiod pathway. It is well established in *Brachypodium*^48^ that the link between the phytocromes and photoperiod control is not direct. In *Arabidopsis*, upon receiving the last signal, the phytochrome photoreceptors interact with other proteins, regulating downstream processes, including TFs of the PHYTOCHROME INTERACTING FACTORS (PIF) family. PIFs are central components of light and temperature signal transduction pathways^45^, acting as transcriptional regulators and repressors of phytochrome signaling^44^. PIFs seem to play a role in response to light quality in our experiment as well. At least two phytochrome interacting factor genes were DE in this study, upregulated under fluorescent light in Price (HORVU5Hr1G102240) and WA1614-95 (HORVU5Hr1G011780). Other DE genes upregulated in F in the sensitive genotypes encode for protein kinases (HORVU5Hr1G001730 in both Price and WA1614-95), other kinase-encoding transcripts in each of those two genotypes, or a RING/U-box superfamily protein (HORVU3Hr1G087720), which may act as an E3-ubiquitin protein ligase. Kinases may phosphorylate residues on PIFs, whereas ubiquitin ligases are involved in light-dependent polyubiquitination of PIFs, leading to their degradation^44^.

Lately, it has become clear that the complex ELF3/LUX (part of the evening complex of the circadian clock), is instrumental in light-mediated repression of *PPD1* genes in rice^49^, Brachypodium^48,50^ and wheat^51^. Apparently, the repression is caused by binding of the ELF3/LUX protein complex to the *PPD1* promoter. We have found differential expression for *HvLUX* in two genotypes, and no significant differences for *HvELF3*. However, we cannot rule out their effect because in our study samples were taken at midday, probably before their peak expression, at the end of the day. Moreover, we do not know whether light quality might have an effect on the stability of ELF3 and LUX or their complex.

An important result of this study is the presence of phenotypic variation of barley in response to light quality. Could this variation be adaptive? Light features, namely duration (photoperiod), intensity, and quality, affect plant adaptation. The effects of photoperiod are well known, and are among the main drivers of annual crop adaptation. Light intensity and quality effects on plant morphological adaptation have also been reported^52,53^. There are also reports of light quality affecting tree growth (Ref.^54^ and references therein). Interestingly these authors found that there is wide natural variation of responses to light quality. This is caused by adaptation to changes in the duration of periods under low solar angles, which depend on latitude, and by variation of overcast conditions (both causing variable R:FR ratios). They suggested that trees from high latitudes are more sensitive to light quality, compared to those from lower latitudes.

Barley spread from its domestication cradle at 35°–40° N to latitudes beyond 60° N. It started its cultivated history as an autumn-sown crop but, later, expanded its niche to spring sowings, thanks to well-known adaptation mechanisms like insensitivity to day length^55^. The barley lines tested in our experiment are autumn sown, and much of their growth period occurs in winter, the period of the lowest solar angle. It is conceivable that annual crops also took advantage of mutations to optimize their growth in regions with light quality features different from the ones found at their low latitude center of origin.

Adaptations to light-related factors for crop species other than day length have been largely overlooked. If factors related to light quality and/or intensity underlie light adaptation of crops, this area deserves urgent attention. Climate change is already causing latitudinal shifts of variety distribution, and plant breeders should know how to cope with possible light-related genetic effects other than photoperiodic response^56^.

It is difficult to pinpoint which part of the light spectrum was causing the contrasting genotypic responses observed in our experiment. This will be the subject of future research. We can speculate that if latitudinal adaptation was the underlying factor, it is possible that, in high latitude regions, with very long favorable seasons, winter and facultative barleys may grow too fast for good agronomic performance with either *PPD-H1* allele. In this case, a mechanism to dilute the photoperiodic response by reducing *PPD-H1* expression would slow down development, promoting the growth of more tillers, or spikes with more florets, thus enhancing yield-supporting structures.

To the best of our knowledge, this study, and our previous article^8^, are among the first reports revealing genetic variability in response to light quality in a cereal crop, and opens up new avenues for future research. This area of research may have practical implications. The strategy known as *speed breeding* has become widely used in the plant breeding industry^57^, speeding up generation time by the combined use of light quality and light duration fine-tuned to hasten plant development. In particular, further research is needed to pin down the underlying molecular bases of the abatement of the photoperiodic response, and to find out whether this genetic variability has an adaptive (latitudinal?) role under natural conditions, which would have clear repercussions on crop breeding.

## Material and methods

### Plant material and phenotyping

Price and WA1614-95 were part of the US CAP project^58,59^; Esterel is a French cultivar from the company Secobra (Table S1). Esterel and Price are winter varieties (with an active *HvVRN2* allele and a winter *HvVRN1* allele, and must be sown in autumn), whereas WA1614-95 is a facultative variety (with a winter allele in *HvVRN1*, but lacks an active *HvVRN2* allele, and can be sown either in autumn, winter, or spring). These genetic constitutions indicate that, to have a timely progression towards flowering, Esterel and Price need a long vernalization period, whereas WA1614-95 would need, at most, a short vernalization period. All three varieties were equally vernalized, to avoid differences in growth that would result from insufficient vernalization. The experiment was carried out in 2016 at the phytotron facilities of the Agricultural Research Institute of the Hungarian Academy of Sciences, Martonvásár (Hungary), using Conviron PGR-15 growth chambers (Conviron Ltd., Canada). Seeds were pre-germinated in peat blocks for 1 week at room temperature, and then fully vernalized (5 ± 2 °C for 52 days under 8 h light/16 h night, low-intensity metal-halide light bulbs). After vernalization, all lines were at a similar developmental stage (Z11-Z12 of Zadoks’s scale^15^). Subsequently, the plants were transferred to pots (12 × 18 cm) holding approximately 1.5 kg of a 3:2:1 mixture of garden soil, compost and sand; and distributed to two growth chambers, either with Sylvania cool white fluorescent light or Tungsram HGL-400 metal halide light bulbs. Both are broad-spectrum light sources, with differences in their spectral composition at the photosynthetically active region (400–700 nm, Fig. S4): fluorescent lights are rich in the blue (430–450 nm), green-yellow (520–630 nm) regions, whereas metal halide has a more balanced spectrum. The main differences in spectra occur at the blue, green-yellow and far-red regions. Absolute photon irradiance was measured at the top of the plant canopy with a USB400-UV-VIS Spectrometer (Ocean Optics, USA). Spectral data was obtained for the interval 350–873 nm every 0.21 nm, and calculated as spectral photon distribution and the summation over the interval as photon flux.

The height of the lamps in the fluorescent light chamber was adjusted once per week to 1.4 m above the canopy, to match the light intensity of the metal halide light chamber, in which the lights were set at a fixed height. The conditions in both chambers were set to long photoperiod (16 h light/8 h night), and 18 ± 1 °C constant temperature, and light intensity of 250 µmol m^–2^ s^–1^. Temperature was continuously monitored through an air-sampling channel, located in the middle of the cabinet, and moved weekly to canopy level. This system of temperature control eliminated the possibility that plants experienced different temperatures at both chambers.

Four pots with one seedling each per genotype and treatment were used for the phenotypic measurements, reported in^8^. Plant development was monitored twice a week by counting leaf and tiller number, measuring plant height, and checking for first node appearance (plant developmental stage 31, or DEV31) and appearance of the awns just visible above the last leaf sheath (DEV49). Another 20 plants per genotype and treatment were planted in groups of 5 per pot (same size), and were used for destructive samplings to record apex development stage (n = 4), and for gene expression studies (n = 3). Dissection of the main tiller’s apex was carried out 23 days after the end of the vernalization period in 4 plants per variety and treatment. Phenotyping consisted of recording apex stage following the Waddington’s scale^60^.

### RNA extraction and transcriptome sequencing

Sampling for RNA-seq took place three days before the examination of the apices shown in Fig. 1. At that time, all three varieties had either clearly started the reproductive phase (Esterel), or were close to that moment (Price and WA1614-95). We measured gene expression in three biological replicates of each genotype grown under the two light conditions. Each biological replicate was a pool of the last expanded leaves from the main tillers of two different plants. Leaves were sampled in the middle of the light cycle (8 h after lights were turned on), 20 days after the end of the vernalization period, and were immediately frozen in liquid N_2_. Samples were collected by two groups of 3 people, working in parallel, to minimize sampling time.

RNAseq was performed by Novogene (HK) Co. Ltd. (China). Eighteen barcoded libraries were multiplexed and sequenced, 2 × 150 bp paired-end reads, in an Illumina HiSeqTM 2500 sequencer, yielding on average 50 Million reads per sample. The whole dataset consisted of 18 samples, i.e. 3 biological replicates, from 3 varieties and 2 light conditions.

Sequencing yield, read curation, assembly, and unigene calling were performed following the pipeline presented in Fig. S5. Raw reads were processed with Illumina CASAVA v1.8 (Illumina, USA). Low-quality reads (reads with more than 50% low-quality base, Q ≤ 20) were removed. Reads from the three genotypes were jointly assembled de novo, with the software Trinity^61^. The assembled transcripts had a median length of 366 bp. Raw transcripts of all three genotypes were combined, followed by a step of hierarchical clustering. Then, the longest transcripts were kept and unigenes were called with Corset v1.05 (–m 10) to remove redundancy^62^. The median unigene length was 779 bp. Raw sequence reads and assembled transcripts have been submitted to the European Nucleotide Archive (project PRJEB35759).

### Quantification of gene expression and differential expression analysis

A reference-based approach was taken using all CDS isoforms annotated in barley reference IBSCv2^63^. In order to do this, we mapped the filtered reads against the reference sequences and quantified transcript abundance as Transcripts Per Million (TPM) using Kallisto^64^. We used the R functions ‘heatmap’ and ‘hclust’^65^, to cluster the gene expression patterns from the experimental replicates. The resulting dendrograms were used to assess the expression quantification.

For compatibility, the gene models were also mapped to genes annotated in the current MorexV3 genome sequence^66^. This was done by lift-over with Liftoff and Barleymap and default parameters^67,68^. Note that in some cases this produced 1-to-many mappings, where one gene in the old annotation corresponds to several genes in MorexV3. These mappings are available at https://github.com/eead-csic-compbio/eead-csic-compbio.github.io/tree/master/data .

Homogeneity of biological replicates was tested with Pearson correlation coefficients plotted with R package “corrplot”^69^. Outliers were called with function Boxplot from R package “car”^70^.

As mentioned above, the metal halide light treatment was considered as control. Thus, DE genes are expressed in terms of being up- or down-regulated under fluorescent light. We used Sleuth^71^ for calculating differential expression (DE) of the genes. Several DE contrasts were performed (metal vs fluorescent for each genotype, and comparisons between pairs of genotypes in the same light quality condition, see Supplementary Datasets S1-S7). DE isoforms were detected using False Discovery Rate (FDR) adjusted p-values (named “q-values”). Initially, only genes with q-value lower than 0.01 were considered differentially expressed. However, this cut-off was relaxed for GO and cluster analysis (see below, * and ** will symbols are used to indicate which cutoff was used). We produced Venn diagrams summarizing the intersection of individual genes affected by light quality in the three genotypes.

DE genes were also identified for comparisons between genotypes in the M light treatment. Price and WA1614-95 developed slower than Esterel at the M treatment^8^. Also, Esterel was intrinsically earlier than the other two genotypes, provided vernalization was completed. As sampling time was common for all three varieties, they reached that point at different growth stages. Esterel in the M treatment was clearly more advanced than any other genotype/treatment combination. Therefore, DE genes between Esterel and the other two varieties in the M treatment are indicative of different transcription patterns due, at least partly, to the developmental stage. These genes were subsequently identified and used to mask developmental changes between the M and F treatments for each variety, and thus increase the chance of detecting those genes primarily responding to light quality.

### Gene ontology enrichment analysis

Gene Ontology (GO) terms for MorexV3 gene models were retrieved from https://search.datacite.org/works/10.5447/ipk/2021/3 and transferred back to IBSCv2 genes using the lift-over mappings described above. GO enrichment tests for Biological Process, Cellular Component and Molecular Function were subsequently performed with R BioConductor TopGO (release 3.14)^72^ using Q-value cutoffs of 0.05 and 0.01. For interpretation lists of GO terms were reduced and plotted with release 3.14 of R Bioconductor package rrvgo^73^.

### Motif discovery in upstream sequences

Upstream promoter sequences of selected DE genes were extracted using the RSAT Plants server at http://plants.rsat.eu^74^. We followed the motif discovery protocol described in^75^, available at https://github.com/rsa-tools/motif_discovery_clusters, which analyzes in parallel upstream sequences with distinct boundaries ([− 1500, + 200], [− 500, + 200], [− 500, 0] and [0, + 200], with coordinates around Transcription Start Sites). Briefly, for each set of promoters analyzed, 50 clusters of the same size, made by random picking barley upstream sequences of the same length and coordinate range, were used as negative controls for assessing the significance of motifs found. The resulting motifs were compared to motifs annotated in the footprintDB database^76^. Note that MorexV3 sequences were used. DE genes which could not be mapped to this reference annotation were left out of the analysis.

### Statement of compliance with guidelines and regulations

Barley is a common crop grown worldwide. The plant specimens used in this experiment are commercial barley cultivars, which are free to use for research and breeding purposes. They are not subject to the Convention on Biological Diversity, the International Treaty on Plant Genetic Resources for Food and Agriculture, or the Convention on the Trade in Endangered Species of Wild Fauna and Flora. All methods were carried out in accordance with relevant guidelines and regulations.

### Supplementary Information

Supplementary Information 1.

Supplementary Information 2.

## Data Availability

The sequence reads generated for this study can be found in the European Nucleotide Archive (ENA) at https://www.ebi.ac.uk/ena/browser/view/PRJEB35759.

## Abbreviations

DE: Differentially expressed
DEV: Developmental stage
F: Fluorescent
LD: Long day
M: Metal halide
TF: Transcription factor
