# Supplementary material for: Diversity of gene expression responses to light quality in barley
Source: Sci Rep. 2023 Oct 10;13:17143. doi: 10.1038/s41598-023-44263-8 (PMC10564772; doi:10.1038/s41598-023-44263-8)
Supplement: Supplementary file 1 — Supplementary Information 1. [file 41598_2023_44263_MOESM1_ESM.pdf]

Supplementary Information

Title: Diversity of gene expression responses to light quality in barley

Authors: Álvaro Rodríguez del Río, Arantxa Monteagudo, Bruno Contreras-Moreira, Tibor Kiss, Marianna Mayer, Ildikó Karsai, Ernesto Igartua, Ana M. Casas

Supplementary Table S1. List of the barley genotypes examined and allelic variants for the major genes of flowering time.

**Variety name**	**Origin**	**Row number**	**Growth habit**	***HvVRN1*[a]**	***HvVRN2*[b]**	***HvFT1*[c]**	***PPD-H1*[d]**	***HvFT3*[e]**
Price	USA	6	Winter	vrn1	VRN2	TC	PPD1	PPD2
WA1614-95	USA	6	Facultative	vrn1	vrn2	AG	PPD1	ppd2
Esterel	France	6	Winter	vrn1	VRN2	TC	PPD1	ppd2

[a] Alleles based on the size of intron 1 (Szűcs et al., 2007; Hemming et al., 2009)

[b] Presence/absence of HvZCCT (Yan et al., 2004)

[c] Alleles based on two SNPs in intron 1, as reported previously (Yan et al., 2006).

[d] Alleles based on SNP22 (Turner et al., 2005)

[e] Presence/absence of PPD-H2 (Faure et al., 2007)

Supplementary Table S2. Predicted DNA motifs and sites of differentially expressed transcription factors among up- (U) and down- (D) regulated genes under F light in both Price and WA1614-95. Predictions made by footprintDB based on similarity of encoded protein sequence (Sebastian and Contreras-Moreira, 2014). Sites scanned at RSAT Plants with matrix-scan-quick-quick with pval<=0.0001 (Markov=1, Santana-García et al., 2022).

transcription factor / annotation	predicted motif(s)	D sites (weight)	D genes	U sites (weight)	U genes
HORVU3Hr1G095090<br><br>*FOREVER YOUNG FLOWER* (*FYF*) caused a significant delay of senescence… by suppressing the ethylene response….<br><br>*FYF* was found to be highly expressed in young flowers prior to pollination and was significantly decreased after pollination...	AGL20<br>(SOC1)<br><br>AGL1, SHP1<br><br>AGL42<br>(FYF)	10 (8.7)	5	12 (12.4)	11
HORVU4Hr1G052490	MYB62	1 (7.6)	1	5 (9.3)	4
HORVU3Hr1G095880	NAC83	4 (6.1)	4	3 (6.1)	2
HORVU2Hr1G034420	WRKY3	4 (7.8)	3	10 (10.9)	8
HORVU5Hr1G093150	ATHB9	5 (5.5)	4	2 (6.3)	2
HORVU3Hr1G087720	-				

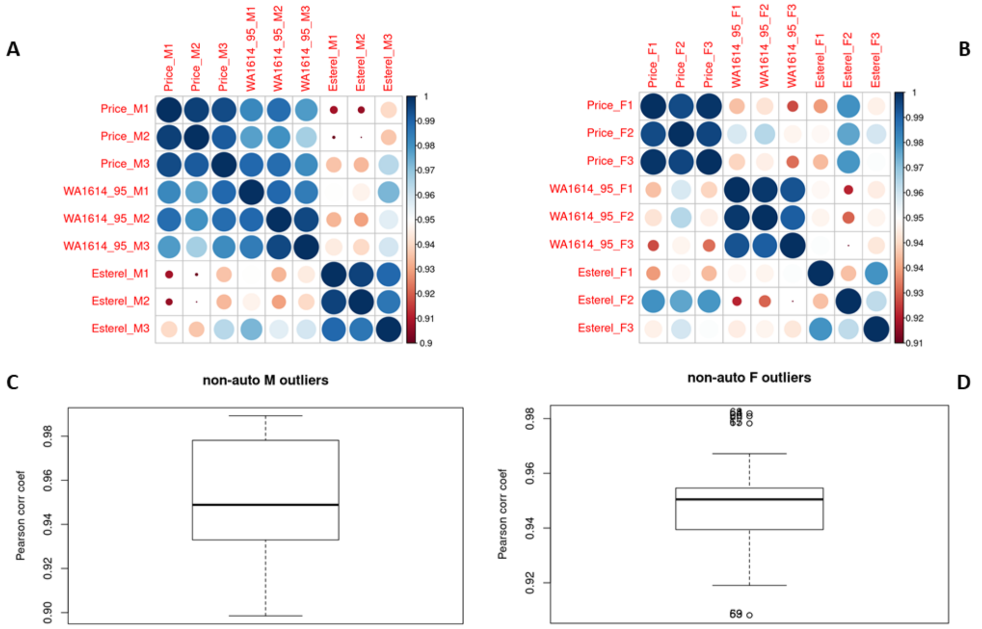

Supplementary Figure S1. Biological replicates of transcript abundances obtained after mapping reads to Morex CDS sequences. The top plots show correlation matrices of gene expression under metal halide bulbs (A) and fluorescent light (B) obtained with R package corrplot. The bottom box plots summarize both distributions of correlation coefficients after excluding values from the main diagonal (auto-correlations). While there are no outliers called in C, the fluorescent boxplot in panel D shows several data as outliers (“Esterel_F2 WA1614_95_F3", "WA1614_95_F3 Esterel_F2", "Esterel_F2 Price_F1", "Esterel_F2 Price_F2", "Esterel_F2 Price_F3", "Price_F1 Esterel_F2", "Price_F2 Esterel_F2" and "Price_F3 Esterel_F2"). These boxplots were computed with R package car.

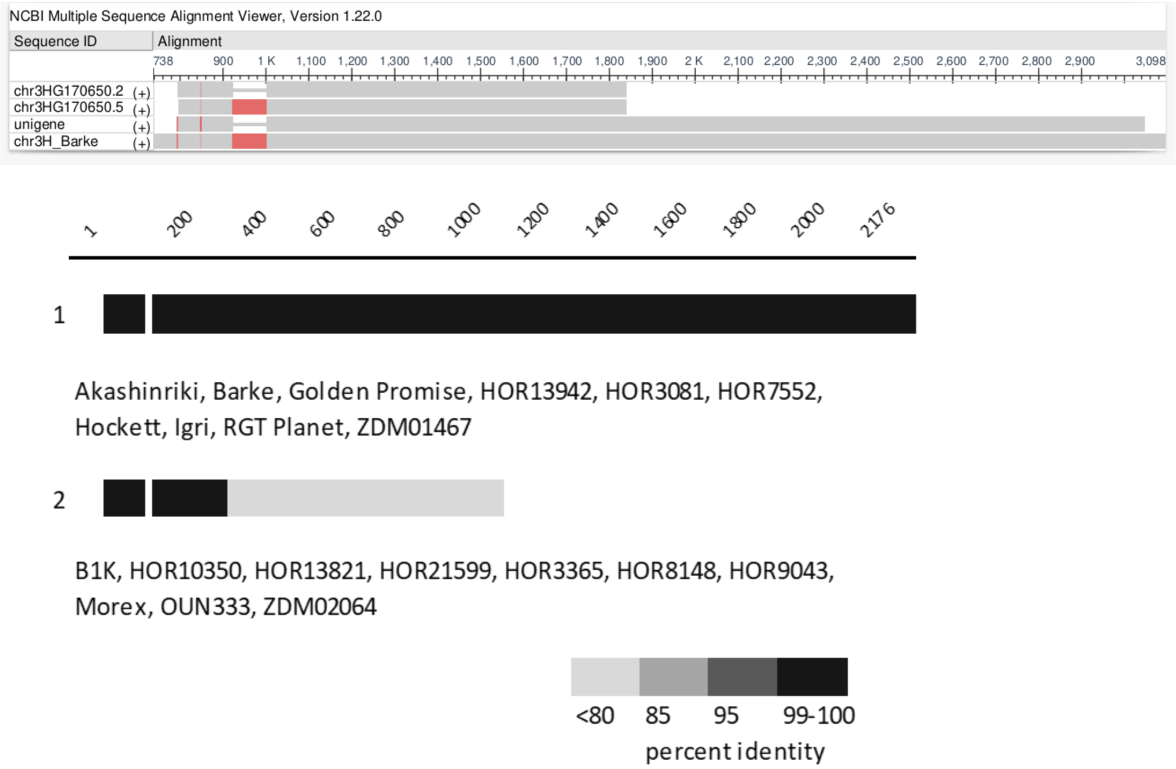

Supplementary Figure S2. Sequence analysis of a unigene referenced to transcript HORVU3Hr1G111550.2 (Cluster-39887.75322-c81701-g1i5, length=2176b). This transcript was found to be differentially expressed in opposite directions in the insensitive and sensitive barley genotypes. Top) Multiple sequence alignment of the differentially expressed unigene, two isoforms of gene chr3HG170650 from the BaRT2v18 high-quality annotation (Coulter et al. 2022) and the genomic sequence of barley cultivar Barke. The ruler shows internal coordinates of the interval chr3H:602169386-602173433 of Barke. Bottom) Idealized representation of the alignment results of the unigene against pangenome barley genotypes (Jayakodi et al. 2020). 1. Genotypes with a perfect match in two split alignments of length 126 and 2048 bp covering the whole transcript; 2. Genotypes with a perfect match in two short alignments of 126 and 257 bp. The coordinates corresponding to Morex v3 are 3H: 605827854-605828867. The figure summarizes the results of 20 BLASTN searches at GrainGenes (Yao et al. 2022).

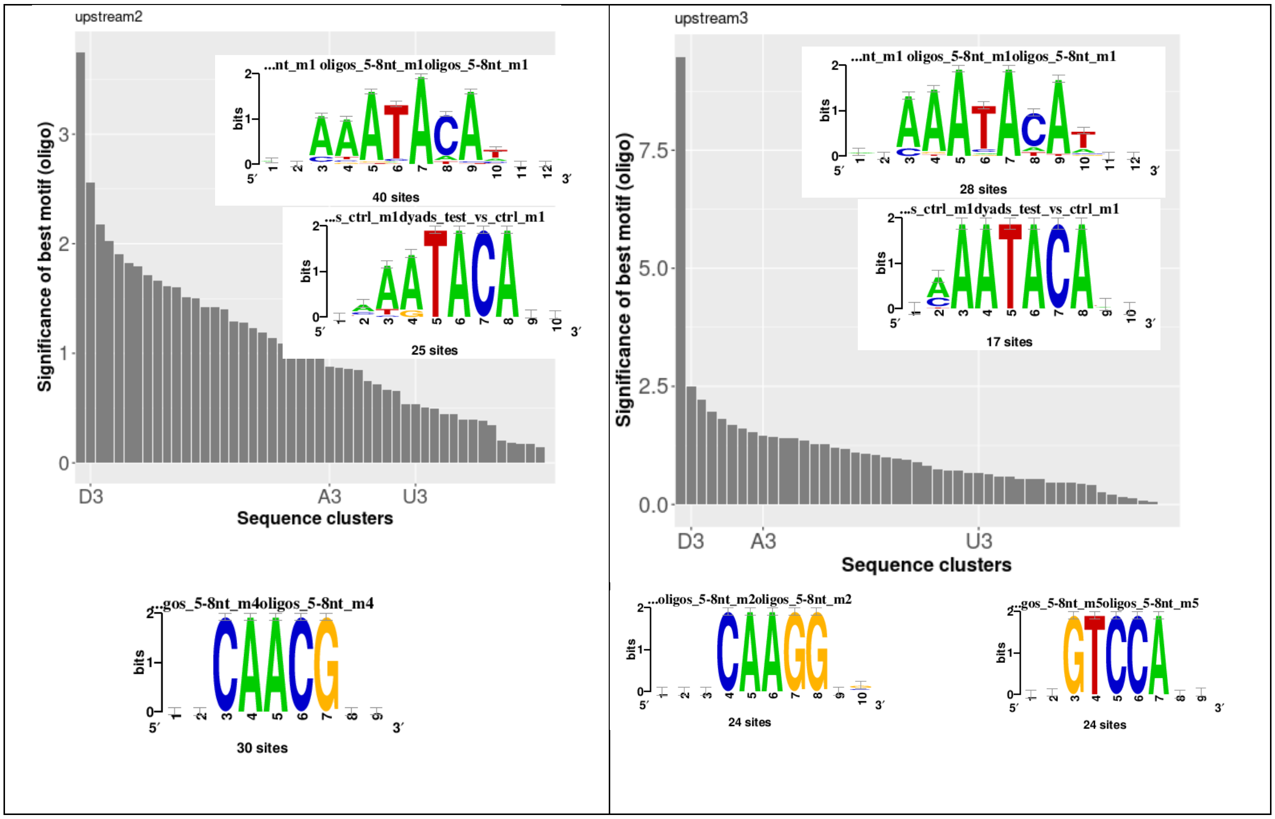

Supplementary Figure S3. Motif discovery within upstream regions of the 50 genes found to be differentially expressed in F light in genotypes Price and WA1614-95. Results are shown for [-500, +200] (left) and [-500, 0] (right) upstream regions. Frequencies of occurrences of motifs in those regions (labelled in the X axis) are compared with occurrence of the same motifs in the corresponding upstream regions of random sets of 50 MorexV3 genes, taken as negative controls. The barplots show the significance of the best DNA motif found in each sequence cluster, produced by the underlying discovery algorithms (oligo-analysis for single words and dyad-analysis for paired words). The Y axis depicts the $-\log_{10}$ of the probability of finding specific motifs in each set of 50 genes. Labelled bars in the X axis indicate the significance of motifs found on 19 down-regulated (D3), 31 up-regulated (U3) and all (A3) differentially expressed genes. Unlabeled bars correspond to negative controls, obtained by randomly sampling sets of 50 MorexV3 genes and cutting the corresponding upstream regions. The sequence logos above describe the most significant motif discovered by both algorithms on down-regulated upstream sequences. Other motifs found are shown below for completeness, but are not discussed in the main text due to their low significance and smaller number of sites.

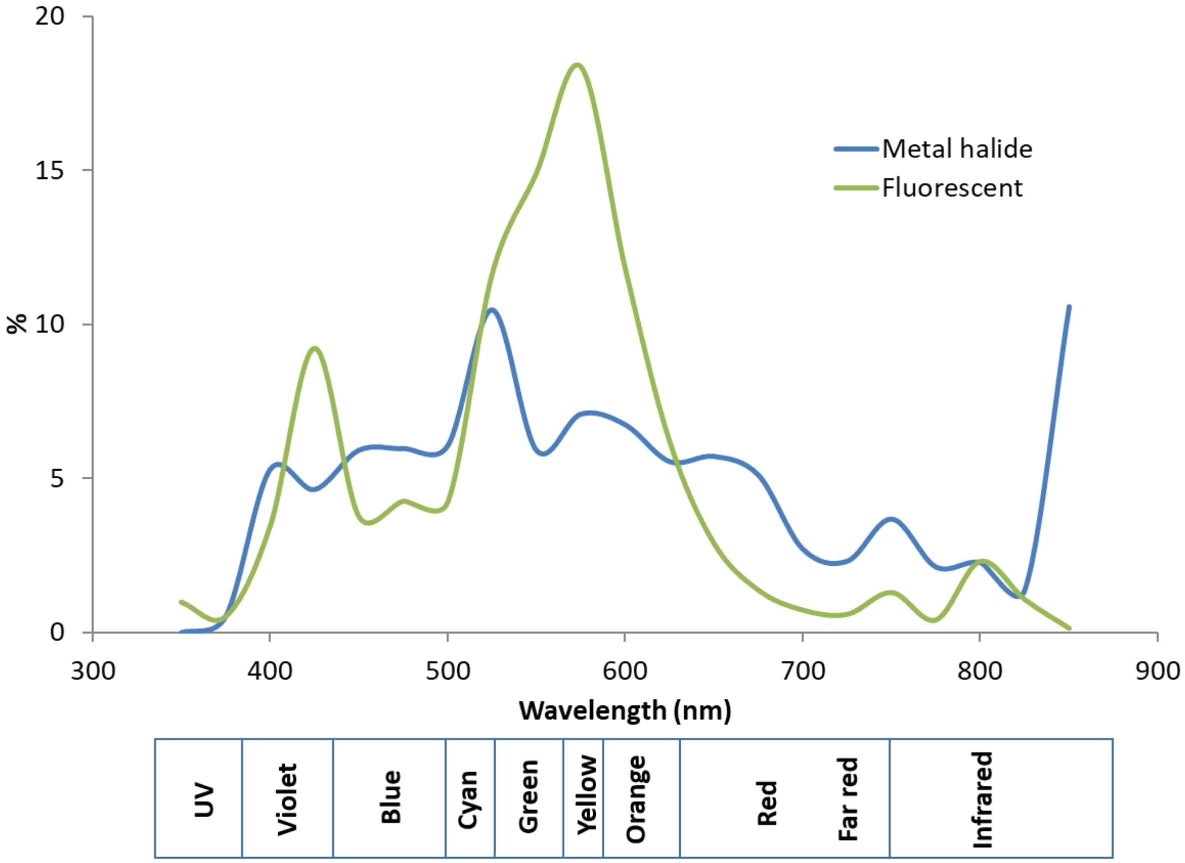

Supplementary Figure S4. Spectral profile of fluorescent (solid line) and metal halide (dotted line) light bulbs measured as proportion of irradiance (%) in intervals of 50 nm in the photosynthetically active region (400-870 nm).

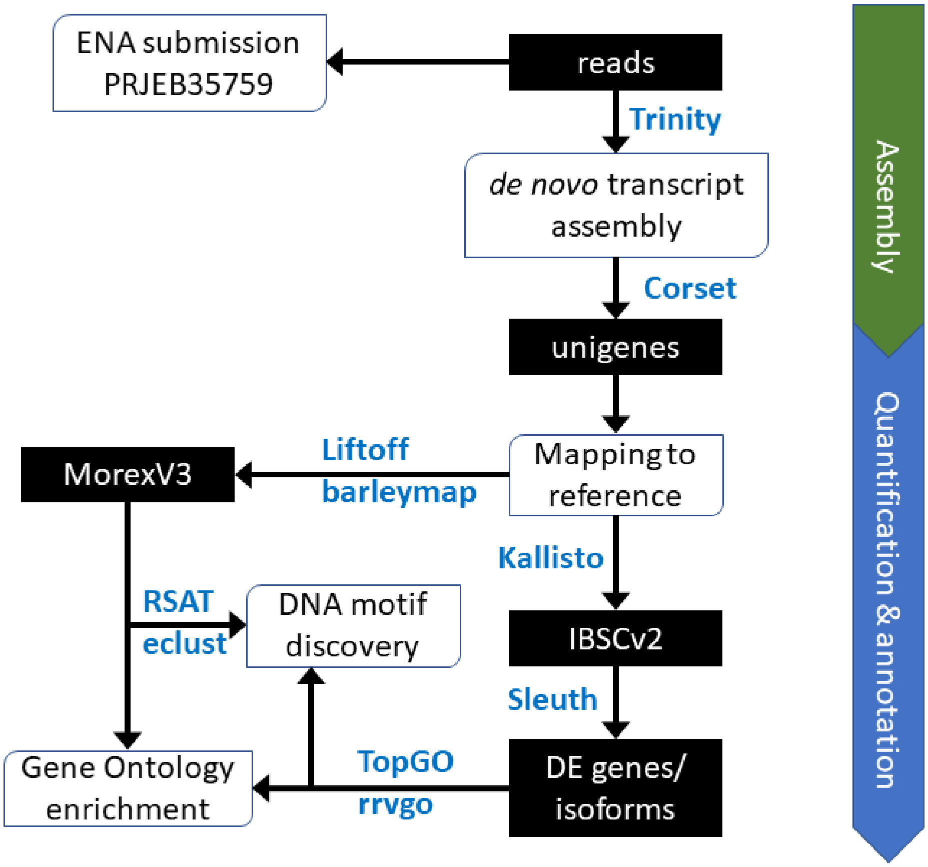

Supplementary Figure S5. Pipeline of the RNA-seq analysis.

References

Coulter, M., Entizne, J.C., Guo, W., Bayer, M., Wonneberger, R., Milne, L., Schreiber, M., Haaning, A., Muehlbauer, G.J., McCallum, N., Fuller, J., Simpson, C., Stein, N., Brown, J.W.S., Waugh, R., Zhang, R. (2022). BaRTv2: a highly resolved barley reference transcriptome for accurate transcript-specific RNA-seq quantification. *Plant J.* 111, 1183-1202. doi:10.1111/tpj.15871

Faure, S., Higgins, J., Turner, A., and Laurie, D. A. (2007). The *FLOWERING LOCUS T*-like gene family in barley (*Hordeum vulgare*). *Genetics* 176, 599–609. doi:10.1534/genetics.106.069500.

Hemming, M. N., Fieg, S., James Peacock, W., Dennis, E. S., and Trevaskis, B. (2009). Regions associated with repression of the barley (*Hordeum vulgare*) *VERNALIZATION1* gene are not required for cold induction. *Mol. Genet. Genomics* 282, 107–117. doi:10.1007/s00438-009-0449-3.

Jayakodi, M., Padmarasu, S., Haberer, G., Bonthala, V.S., Gundlach, H., Monat, C., et al. (2020). The barley pangenome reveals the hidden legacy of mutation breeding. *Nature* 588:284–289. doi:10.1038/s41586-020-2947-8.

Santana-García, W., Castro-Mondragon, J.A., Padilla-Gálvez, M., Nguyen, N.T.T., Elizondo-Salas, A., Ksouri, N., et al. (2022). RSAT 2022: regulatory sequence analysis tools. *Nucleic Acids Res.* 50, W670–W676. doi: 10.1093/nar/gkac312.

Sebastian, A., and Contreras-Moreira, B. (2014). footprintDB: a database of transcription factors with annotated cis elements and binding interfaces. *Bioinformatics* 30, 258–265. doi:10.1093/bioinformatics/btt663.

Szűcs, P., Skinner, J. S., Karsai, I., Cuesta-Marcos, A., Haggard, K. G., Corey, A. E., et al. (2007). Validation of the VRN-H2/VRN-H1 epistatic model in barley reveals that intron length variation in *VRN-H1* may account for a continuum of vernalization sensitivity. *Mol. Genet. Genomics* 277, 249–261. doi:10.1007/s00438-006-0195-8.

Turner, A., Beales, J., Faure, S., Dunford, R. P., and Laurie, D. A. (2005). The pseudo-response regulator *Ppd-H1* provides adaptation to photoperiod in barley. *Science* 310, 1031–1034. doi:10.1126/science.1117619.

Yan, L., Loukoianov, A., Blechl, A., Tranquilli, G., Ramakrishna, W., SanMiguel, P., et al. (2004). The wheat *VRN2* gene is a flowering repressor down-regulated by vernalization. *Science* 303, 1640–1644. doi:10.1126/science.1094305.

Yan, L., Fu, D., Li, C., Blechl, A., Tranquilli, G., Bonafede, M., et al. (2006). The wheat and barley vernalization gene *VRN3* is an orthologue of *FT. Proc. Natl. Acad. Sci.* 103, 19581–19586. doi:10.1073/pnas.0607142103.

Yao, E., Blake, V.C., Cooper, L., Wight, C.P., Michel, S., Cagirici, H.B., Lazo, G.R., Birkett, C.L., Waring, D.J., Jannink, J.L., Holmes, I., Waters, A.J., Eickholt, D.P., Taner, S.Z. (2022). GrainGenes: a data-rich repository for small grains genetics and genomics. *Database*, 2022, baac034, https://doi.org/10.1093/database/baac034
